# New materials and devices for preventing catheter-related infections

**DOI:** 10.1186/2110-5820-1-34

**Published:** 2011-08-18

**Authors:** Jean-François Timsit, Yohann Dubois, Clémence Minet, Agnès Bonadona, Maxime Lugosi, Claire Ara-Somohano, Rebecca Hamidfar-Roy, Carole Schwebel

**Affiliations:** 1 Medical Polyvalent Intensive Care Unit, University Joseph Fourier, Albert Michallon Hospital, BP 217, 38043 Grenoble Cedex 9, France; 2 University Joseph Fourier, EA U823, Albert Bonniot Institute, 38706 La Tronche Cedex, France

## Abstract

Catheters are the leading source of bloodstream infections for patients in the intensive care unit (ICU). Comprehensive unit-based programs have proven to be effective in decreasing catheter-related bloodstream infections (CR-BSIs). ICU rates of CR-BSI higher than 2 per 1,000 catheter-days are no longer acceptable. The locally adapted list of preventive measures should include skin antisepsis with an alcoholic preparation, maximal barrier precautions, a strict catheter maintenance policy, and removal of unnecessary catheters. The development of new technologies capable of further decreasing the now low CR-BSI rate is a major challenge. Recently, new materials that decrease the risk of skin-to-vein bacterial migration, such as new antiseptic dressings, were extensively tested. Antimicrobial-coated catheters can prevent CR-BSI but have a theoretical risk of selecting resistant bacteria. An antimicrobial or antiseptic lock may prevent bacterial migration from the hub to the bloodstream. This review discusses the available knowledge about these new technologies.

## Introduction

Central venous catheters (CVCs) are inserted in approximately half of all patients in the intensive care unit (ICU). In Europe, the incidence density of catheter-related bloodstream infections (CR-BSI) ranges from 1 to 3.1 per 1,000 patient-days [1]. CR-BSIs were associated with an attributable mortality of 0% to 11.5% [2] and an additional stay length of 9-12 days [3,4].

In contrast to other nosocomial infections, CR-BSI has many device-related risk factors. Consequently, prevention should be possible, provided that rigorous policies are implemented. Specific education and training of healthcare workers in CR-BSI prevention and continuous implementation of unit-based quality-improvement programs are essential. We discuss the potential usefulness of new technical developments and put these into perspective according to available recommendations.

### Mechanisms of infection

Colonization of the catheter occurs via two main pathways: the extraluminal route and the intraluminal route. Colonization of short-term CVCs (< 15-20 days) occurs predominantly from the skin puncture site, whereas colonization of long-term CVCs is usually related to intraluminal bacterial spread from a contaminated hub [5]. In both cases, the source of the micro-organisms is the patient's own commensal skin flora. Accordingly, *S. epidermidis *is responsible for 40-50% of episodes, followed by *S. aureus *(10-20%). Gram-negative bloodstream infection, especially *Pseudomonas aeruginosa, Stenotrophomonas *sp., and *Acinetobacter baumannii*, are recovered in one-third of cases. *Candida *sp. are recovered in 3-10% of cases.

Biofilm formation on the inner and outer surfaces of the catheter contributes to the development of CR-BSI. A biofilm is a complex structure formed by bacteria that have attached to an artificial surface or dead tissue. Bacterial attachment to the catheter surface begins within 24 hours after catheter insertion. The bacteria proliferate and secrete a polysaccharide matrix, which provides a medium for the attachment of additional organisms. Constitution of a biofilm is virtually inevitable but does not necessarily lead to clinical manifestations of infection, probably because the bacteria contained in the biofilm are characterized by slow growth and limited virulence [6]. Clinical biofilm infection is typically resistant to antimicrobials, not only because the antimicrobials cannot penetrate into all the biofilm layers, but also because the organisms grow slowly and may be resistant to immune defence mechanisms.

The pathogenesis of fibrin sheath formation from the biofilm is poorly understood. According to the best available evidence, biofilm formation is the first event. Subsequently, fibrin and many other molecules, such as laminin, collagen, and even muscle cells, convert the film to a mature sheath [7]. Metallic cations, such as magnesium, calcium, and iron, may stabilize the biofilm and contribute both to its development and bacterial growth [8]. Catheter thrombosis on the fibrin sheath may be facilitated by platelet activation, decreased levels of protein C and antithrombin III, hyperfibrinogenemia, and homocysteine elevation. Clinical evidence suggests that the risk of thrombosis may increase with the severity of the infection, the occurrence of bacteremia, and CR-BSI [9-11].

### Diagnosis of catheter infections

The diagnostic methods that are currently accepted worldwide must be borne in mind, because differences in the methods used clearly introduced bias in trials on preventive strategies. In current French recommendations for patients with BSI, a positive quantitative [12,13] or semiquantitative [14] catheter culture is needed to determine that the catheter caused the BSI. Alternatively, simultaneous comparative quantitative blood samples [15] or the difference in time to positivity of blood from the hub and peripheral blood [16] should be determined. However, the Centers for Disease Control definition of central-line associated bloodstream infection (CLABSI) [17], which does not take catheter culture results into account, often is used as an acceptable surrogate. This definition requires a single blood culture positive for a pathogen (or two cultures positive for a common commensal) not recovered from any nonblood cultures during the 3 days preceding and 7 days following. This definition is insufficiently accurate [18,19]. Considerable variability occurs among experts [20,21] and hospitals [18] in the classification of infections as CLABSI or secondary bacteremia. In addition, this definition is obviously dependent of the number of blood cultures performed before introducing new antimicrobials and the number of nonblood cultures performed to look for an infectious focus responsible for secondary BSI.

### Prevention

A number of published studies have investigated various measures for decreasing the CR-BSI rate [22]. Some of them evaluated multimodal programs to improve general infection control measures when using catheters, such as surveillance, education, and quality management strategies, whereas others tested new biomaterials, antiseptic dressings, and catheter locks.

It should be stated clearly that new biomaterials should be tested and incorporated into routine prevention programs only if they have been proven to further decrease the CR-BSI rate below the value obtained when all the basic guidelines are implemented. We will now discuss these basic guidelines before focusing on the potential benefits of new biomaterials.

### Basic guidelines for prevention

#### Catheter insertion

##### Sterile barrier precautions and skin antisepsis

Although the usefulness of full barrier precautions outside the ICU was challenged recently [23], the standard of care for CR-BSI prevention must continue to include the use of sterile gloves, a long-sleeved sterile gown, a mask, a cap, and a large sterile drape during catheter insertion [24]. Aqueous alcoholic hand rubs improve adherence to and tolerance of hand-cleansing rules and are probably at least as effective as surgical hand washing [25].

Chlorhexidine solution is superior to aqueous povidone iodine (PVI) solution for cutaneous antisepsis [26]. Skin disinfection with 2% alcoholic chlorhexidine significantly diminished the number of peripheral vein tips carrying micro-organisms on their surface compared with 70% ethanol alone [27]. One prospective cohort study found no difference in catheter colonization or CR-BSI between 0.5% alcoholic chlorhexidine and 2% aqueous chlorhexidine [28]. After skin disinfection, the decrease in micro-organism counts is slightly greater when the chlorhexidine concentration is increased [29]. A 2% alcoholic chlorhexidine is now available but is much more expensive than the 0.5% alcoholic solution, and convincing evidence that it is superior to the lower concentration is needed. A 5% povidone-70% ethanol solution was superior to regular 10% PVI in decreasing catheter colonization (odds ratio [OR], 0.38; 95% confidence interval [CI], 0.22-0.65) and catheter-related infections (OR, 0.34; 95% CI, 0.13-0.91) [30]. In none of the available studies was alcoholic-PVI less effective than chlorhexidine solutions. However, a single-center randomized trial found a significantly lower rate of catheter colonization (9.7 vs. 18.3 per 1,000 CVC-days) and a trend toward a lower rate of CR-BSI (1.4 vs. 3.4 per 1,000 CVC-days; *P *= 0.09) with a solution containing 0.25% chlorhexidine gluconate, 0.025% benzalkonium chloride, and 4% benzylic alcohol, compared with 5% alcoholic-PVI [31].

Because chlorhexidine is not only for catheter insertion but also for hand hygiene, preoperative skin preparation and bathing extensive use may result in resistance [32]. Octenidine (0.1%) in propranolol isopropyl alcohol compared favourably to ethanol/propranolol in terms of catheter tip colonization (7.9% vs. 17.8%, *P *= 0.009). This new antiseptic preparation needs to be compared with chlorhexidine and/or alcoholic-PVI [33].

#### Catheter insertion site

CVC insertion is required in many critically ill patients. Selection of the insertion site should be based on both the ease and the risks of the procedure. The risks include infection, thrombosis, and mechanical complications. Subclavian access is preferred for infection control purposes, although other factors (potential mechanical complication, thrombosis, and operator experience) should be considered [34,35]. The use of femoral catheters is associated with a higher rate of thrombosis and should probably be restricted to thin patients [36], in whom the rates of mechanical complications (i.e., pneumothorax and hemorrhage) are unacceptably high with other routes. In the ICU, the femoral or internal jugular vein may need to be used, and it should be kept in mind that catheter tunnelling decreased the risk of CR-BSI [37,38].

#### Ultrasound-guided catheter insertion

Ultrasound guidance has been suggested to decrease the risks of failed catheter insertion and immediate complications. When the internal jugular vein is used, this technique may provide benefits [39], such as decreased rates of failed insertion and mechanical complications. In an open-label randomized trial of 900 patients, ultrasound use was associated with decreases in the CR-BSI rate (10.6% vs. 16%, *P *< 0.01) and in the mean number of insertion attempts [40]. When ultrasound equipment is available and the physician is sufficiently well trained in ultrasonography, ultrasound-guided insertion should be considered routinely before considering insertion in the internal jugular vein. The data on femoral and subclavian insertion are encouraging but are insufficient to draw definitive conclusions.

#### Catheter care: replacement, dressings, and tubings

Repeated catheterization may be unavoidable but increases the risk of catheter infection [41]. Given this fact and the results of randomized studies, CVCs that are functioning in patients with no evidence of local or systemic complications should not be replaced routinely. This rule may not apply to Swan-Ganz catheters and arterial catheters, for which the daily risk of infection may increase with the duration of catheter maintenance [42,43]. Physicians and nurses should assess the patient's need for an intravascular catheter on a daily basis.

Semipermeable transparent dressings, which are widely used, allow continuous observation of the skin insertion site and reduce the risk of extrinsic colonization. A gauze dressing is preferred if blood is oozing from the catheter insertion site. Catheter dressings should be changed immediately if they become damp, loosened, or soiled.

The optimal frequency of routine of CVC dressing changes is unknown. The interval between scheduled changes can be safely increased to 7 days in the ICU, provided soiled and loosened dressings are changed immediately [44].

Tubings should be replaced at least every 72 hours. However, tubing replacement only every 4 days, instead of every 2 days, did not increase the rate of CR-BSI [45]. Nevertheless, tubings used to administer blood, blood products, or lipid emulsions (including propofol infusions) should be replaced within 24 hours [46].

Many needleless intravascular connector valves have been introduced into clinical practice to minimize the risk of needlestick injury. After disinfection of the connections, microbial contamination of these systems is lower compared with three-way stopcocks with caps [47]. However, in cohort studies, needleless systems often were still contaminated after the widely used procedure of alcoholic disinfection for 3-5 sec, which is clearly inadequate. Moreover, most needleless systems are opaque, making it impossible to verify that they were properly flushed. These points explain why needleless systems have been repeatedly associated with BSI outbreaks [48-50]. A large epidemiological study in five hospitals strongly suggested that these systems increased the risk of central line-associated infections [51]. However, new silver-impregnated connectors may decrease microbial contamination.

Overall, any excessive manipulation of CVCs independently increases the risk for CR-BSI and must be avoided [52].

#### Impact of continuous quality-improvement programs

A comprehensive unit-based safety program combining staff education, identification of and learning from deficiencies, assignment of a hospital executive to adopt the unit, and implementation of teamwork tools are essential to all general safety programs. It is of course fundamental to translate the evidence into practice by creating a checklist, identifying local barriers to guideline implementation, measuring performance, participating in a global network [53], and ensuring that all healthcare workers are aware of the evidence. The general components of a unit-based safety program for controlling CR-BSIs should be easy to apply.

Practical recommendations for catheter insertion, care, and surveillance always include three general preventive measures: prevention and control of multiresistant bacteria spread, hand hygiene, and surveillance of nosocomial infections. Specific measures to prevent catheter infections can be effective only if they are followed scrupulously. Healthcare workers should first focus on several established methods directed at preventing contamination of the catheter. Suggested bundles should rest on available recommendations and be adapted locally. The most commonly accepted recommendations are: 1) improve adherence to hand hygiene rules; 2) insert catheters via the subclavian route whenever possible; 3) use antiseptic solution containing alcohol; 4) inspect the insertion site daily; 5) immediately change loosened, soiled, or moistened catheter dressings; and 6) immediately remove catheters that are no longer indispensable. These simple recommendations often are violated in everyday practice if the healthcare workers are not reminded of them frequently. Recent cross-sectional surveys still found that they were not routinely followed, particularly outside of the ICU [54,55].

Strong educational efforts designed to obtain the compliance of all healthcare workers with established protocols must be regularly discussed and updated, and continuous surveillance of CVC infection rates with feedback to the staff should be instituted. Importantly, the effects of these educational programs may be sustained if staff members are involved in designing the measures included in the program and if all new nurses, residents, and fellows follow an introductory in-service training program [56]. Simulation-based learning was recently found to be more effective than video training alone to improve residents' skills [57,58] and led to a dramatic decrease in the CR-BSI rate when the rate in the control group was high. In a global safety culture program within the ICU, the identification of a program leader also is a key factor for success.

Between 2002 and 2010, these recommendations were found to be successful in many published studies, most of which were done in the ICU [20,59-62]. In Michigan, a comprehensive approach based on a bundle of care combined with an improvement in the safety culture and teamwork was associated with a dramatic decrease in CLABSIs, from 7.7 to 1.4 per 1,000 CVC-days, in 103 ICUs (1,981 ICU months; 375,757 catheter-days) [60], and this effect was sustained [63]. The intervention was based on five recommended procedures: improved hand washing, use of full-barrier precautions during CVC insertion, skin cleansing with chlorhexidine, avoidance of the femoral site whenever possible, and removal of unnecessary catheters. The before-after design (with potential regression-to-the-mean and Hawthorne effects), very high baseline CLABSI rate, lack of accuracy of the CLABSI definition [18,64], absence of an assessment of compliance with the study measures, and absence of data about the relative importance of the various components of the intervention are important weaknesses that prevent this study from demonstrating a causal relationship between the intervention and the result. Although we believe that the zero-risk concept is unrealistic and both dangerous and counterproductive [65], the implementation of bundles of care (adapted locally) combined with reinforcement of the safety culture is the crucial first step toward improving catheter infection rates.

#### New materials and prevention strategies

##### Antiseptic-impregnated dressings

Even after careful disinfection, regrowth of the skin flora occurs consistently under the transparent dressing, due to the migration of bacteria from the dermis to the epidermis and to the limited efficacy of antiseptic solutions under the superficial skin [66]. Chlorhexidine-impregnated dressings prevent micro-organism regrowth in the epidermis. In a randomized, multicenter assessor-blind trial, we allocated 1,636 patients to catheter dressings with or without chlorhexidine-impregnated sponges. A total of 3,778 arterial and central vein catheters were enrolled (28,931 catheter-days). The use of chlorhexidine-impregnated dressings decreased the risk of major catheter-related infections (0.6 vs. 1.4 per 1,000 catheter-days; hazard ratio [HR], 0.39; *P *= 0.03) and CR-BSI (0.4 vs. 1.3 per 1,000 catheter-days; HR, 0.24; *P *< 0.001) [44]. In adults, the rate of contact dermatitis seen with the chlorhexidine-impregnated sponges was 5.3/1,000 catheters, but no systemic reactions were recorded. In low-birth-weight infants (< 1,000 g), chlorhexidine sponges were associated with a far higher rate of contact dermatitis of 15.3% and therefore should be avoided [67]. New chlorhexidine-impregnated gel dressings were developed recently and have been shown to decrease the cutaneous microflora to a similar extent as the sponges [68]. The clinical efficacy of this new dressing in ICU patients is being tested in a large randomized trial (http://www.clinicaltrial.govNCT 01189682), in which 1,800 of the 1,960 initially planned patients have already been enrolled.

Silver-alginate-coated dressings have been tested in the ICU in neonates who had peripherally inserted central catheters (PICCs). In a 3:1 randomized pilot study involving 100 neonates, the silver-alginate coating was safe and led only to a change in skin color without contact dermatitis [69]. The rate of PICC-associated BSI decreased from 17.2% to 12.4%. This encouraging result warrants a randomized trial with sufficient statistical power to allow a definitive conclusion.

##### Antithrombotic prophylaxis

Both experimental and cohort studies [9,70] suggest a close relationship between catheter thrombosis and infection. Several thrombus proteins increase the adherence of staphylococci and *Candida *spp. to catheters. Thrombus formation on indwelling intravascular catheters is associated with CR-BSI. Experimental work has demonstrated that the formation of a fibrin sheath surrounding the catheter greatly increases catheter colonization [71]. In rats, subcutaneous administration of the low-molecular-weight heparin enoxaparin decreases fibrin sheath formation and the incidence of catheter colonization with biofilm-producing *S. epidermidis *[72]. Recent clinical trials suggest that heparin may reduce catheter-related infections. In a randomized, double-blind study in critically ill children, heparin-bonded catheters decreased the rates of thrombosis (0% vs. 8%, *P *= 0.006) and positive blood cultures (drawn through the catheter; 4% vs. 33%, *P *< 0.0005) [73]. In a double-blind, randomized, controlled trial in neonates, heparin (0.5 IU/mL) added to the total parenteral nutrition preparation decreased all episodes (relative risk [RR] = 0.57, *P *= 0.04) and definite episodes (RR = 0.32, *P *= 0.06) of catheter-related sepsis [74]. Bone marrow transplant patients were randomly assigned to 100 U/kg per day of heparin or saline [75]. They found a significant decrease in the CR-BSI rate in the heparin-treated group (2.5/1,000 CVC-days vs. 6.4/1,000 CVC-days), without any adverse effects. Because most heparin solutions contain preservatives with antimicrobial activity, it is unclear whether a decrease in the CR-BSI rate would be due to decreased thrombus formation or the preservative, or both. The potential benefits of heparin or heparin-coated catheters must be balanced against the risk of heparin-induced thrombocytopenia.

Fibrinolytic solutions may decrease the risk of infection by decreasing biofilm attachment. In a randomized, double-blind, controlled trial, 181 hematology patients with intermediate-term catheters (mean duration, 30 days) were allocated to a catheter lock of 25,000 IU of urokinase or saline for at least 30 minutes, three times per week. The urokinase lock reduced major bloodstream infections (4/82 vs. 13/78) because of an effect limited to coagulase-negative BSIs (1.2% vs. 14.1%; RR = 0.09; 95% CI, 0.01-0.5) and CVC-related thrombosis (1.3% vs. 9%; RR = 0.14; 95% CI, 0.02-0.82) [76]. In long-term dialysis catheters, the urokinase lock decreased the rate of catheter malfunction (22/110 patients vs. 40/115 patients) and significantly decreased the risks of catheter-related bacteremia (0.34 vs. 1.37/1,000 catheter-days; *P *= 0.02) and bacteremia from any cause (HR, 0.3; 95% CI, 0.11-0.85; *P *= 0.02) [77].

##### Antimicrobial-coated or impregnated catheters

The efficacy of catheters whose outer surface is impregnated with chlorhexidine and silver sulfadiazine was tested in many randomized studies in the 1990s. A well-conducted meta-analysis concluded that this technique reduced the risk of CR-BSI (RR = 0.4; 95% CI, 0.2-0.8) in patients with short-term CVCs [78]. It was mainly effective when the median duration of insertion was less than 7 days (median, 6 (range, 5.2-7.5) days; from 4.1% to 1.9%; OR, 0.48; 95% CI, 0.25-0.91) compared with the control catheters (median, 12 (range, 7.8-20) days; from 4.5% to 4.2%; OR, 0.94; 95% CI, 0.58-1.54) [79]. This technique is cost-saving in settings where the incidence of CR-BSI complicating short-term CVCs is very high (more than 3.3 per 1,000 catheter-days) and the average insertion time is less than 8 days.

New chlorhexidine/sulfadiazine-impregnated catheters with a long half-life of impregnation at the internal and external surfaces have now been developed. According to a recent meta-analysis of five randomized, controlled trials, this catheter halves the risk of CR-BSI (OR, 0.51; 95% CI, 0.56-1.00) [80]. However, this meta-analysis unmasked significant heterogeneity across study results, and the pooled CR-BSI rate in the control groups was unacceptably high in two studies (7.2% and 14%). When taking into account only the three studies with acceptable CR-BSI rates, chlorhexidine/silver/sulfadiazine-impregnated catheters failed to significantly decrease the CR-BSI rate (impregnated 8/614 vs. control 9/589 catheters; OR (random effect), 0.852; 95% CI, 0.2-3.6) [81].

Resistance to chlorhexidine-sulfadiazine has not been demonstrated in clinical studies. However, resistance to chlorhexidine has been induced *in vitro *[82]. Rare cases of anaphylactic reaction to the chlorhexidine component of this catheter have been reported [41]. Consequently, chlorhexidine/silver/sulfadiazine-impregnated catheters should be reserved for patients who are expected to require the catheter for less than 8 days and who are admitted to a unit that has high infection rates despite adherence to other strategies, such as maximal barrier precautions and implementation of an educational program. As acceptable incidence rates are between 1 and 3 CR-BSIs per 1,000 catheter-days, the use of such impregnated catheters is not standard practice. Catheters impregnated with oligon, silver zeolite, carbon, and platinum have been tested but have not been proven effective [80].

Catheters impregnated intraluminally and extraluminally with minocycline-rifampin reduce the risk of CR-BSI compared with polyurethane catheters and externally coated chlorhexidine/silver/sulfadiazine-impregnated catheters (OR, 0.23; 95% CI, 0.14-0.4) [83]. The size of the inhibition zone against a reference *S. epidermidis *correlated inversely with the duration of catheter insertion but was greater than 10 mm for a duration of 60 days [84]. Minocycline/rifampin-impregnated catheters decreased the risk of CR-BSI compared with controls (five ICU studies; OR, 0.26; 95% CI, 0.15-0.47) [80]. However, despite eight randomized, controlled trials, no clear conclusion could be drawn regarding the impact of minocycline/rifampin-impregnated catheters on the development of antimicrobial resistance or on the selection of resistant flora. In addition, two recent studies showed an increased risk of *Candida *spp. catheter colonization [84,85]. However, a large prospective 7-year follow-up study of 9,200 catheters (more than 500,000 catheter-days) in a tertiary university cancer center failed to unmask the emergence of bacterial resistance among staphylococcal species [86].

To alleviate concerns about antimicrobial resistance pressure, new 5-fluorouracil (5-FU)-coated catheters were recently developed. The pyrimidine analogue 5-FU is an antimetabolite drug. In concentrations well below those used in cancer therapy, 5-FU has been shown to inhibit the growth of gram-positive and gram-negative bacteria and *Candida *species. In a single-blind, multicenter, noninferiority, randomized trial, catheters coated externally with 5-FU were compared to catheters coated externally with chlorhexidine-silver-sulfadiazine (CH-SS). 5-FU-coated catheters compared favorably to CH-SS-coated catheters in terms of catheter tip colonization (5-FU-coated, 12/419 vs. CH-SS-coated, 21/398 catheters; difference, -2.6% with an upper confidence limit of -0.13%) and CR-BSI (5-FU-coated, 0/65 episodes vs. CH-SS-coated, 2/71 episodes; difference, -2.8%; 95% CI, -10% to +3%) [87].

An extended review of the biocidal efficacies of various antimicrobial coatings was published recently [88].

##### Antibiotic or antiseptic lock solutions

The prophylactic use of systemic antibiotics at the time of catheter insertion has not been proven effective in reducing the incidence of CR-BSI and is strongly discouraged.

Anti-infective lock solutions are intended for catheters that are not used continuously. They are effective in preventing intraluminal contamination. Theoretically, these solutions produce anti-infective concentrations that are sufficient to kill organisms embedded in the biofilm. However, their role in preventing short-term CR-BSI in the ICU is limited to catheters that are not used continuously, such as hemodialysis catheters [41] or PICCs in neonates. A randomized study in critically ill neonates showed an 80% reduction in PICC-related BSI with a vancomycin lock administered for 20 or 60 min twice a day [89]. Prospective screening tests for colonization or infection with vancomycin-resistant organisms in exposed infants were negative.

In a recent meta-analysis, antibiotic lock solutions for long-term hemodialysis catheters prevented one CR-BSI in one of every four patients (95% CI, 4-5) and reduced the rate of catheter removal [90]. However, significant publication bias occurred.

Cationic chelators, such as EDTA (edetic acid) or citrate, have an anticoagulant activity similar to heparin and have been found to enhance the activity of antimicrobial drugs against organisms embedded in the biofilm [8].

Many antimicrobials, such as vancomycin, teicoplanin, daptomycin, gentamicin, cephalosporins, and minocycline, have been tested for lock therapy, with interesting results. However, in our opinion, antibiotic-antiseptic lock solutions for CR-BSI prevention should rely only on molecules that cannot be used for parenteral administration in human patients. Uncontrolled trials have shown that taurolidine, a derivative of the aminosulphonic acid taurine, reduces the risk of CR-BSI associated with hemodialysis catheters and long-term intravenous devices [91,92]. A recent, double-blind, randomized, controlled trial compared interdialytic lock with taurolidine and citrate (1.35% taurolidine and 4% citrate) to heparin (5,000 U/mL) started at catheter insertion in 110 adult hemodialysis patients with tunnelled cuffed intravascular catheters. Taurolidine-citrate solution failed to decrease significantly the risk of CR-BSI (1.4 vs. 2.4 episodes/1,000 catheter-days, *P *= 0.1) but increased the risk of dysfunction or thrombosis requiring thrombolytic treatment [93].

In 407 hemodialysis patients with long-term catheters, Maki et al. recently found in a randomized, assessor-blind study that a novel catheter lock solution with antimicrobial and antithrombotic activity containing 0.24 M (7.0%) sodium citrate, 0.15% methylene blue, 0.15% methylparaben, and 0.015% propylparaben (C-MB-P) compared favorably with heparin in terms of both CR-BSI (0.24 vs. 0.82 per 1,000 catheter-days; RR = 0.29; 95% CI, 0.12-0.7; *P *= 0.005) and loss because of patency failure (0 vs. 4; log-rank, *P *= 0.04) [94]. The impact of this new lock needs to be tested in short-term hemodialysis catheters used in the ICU.

Ethanol lock solutions also have been evaluated [92]. *In vitro*, 2 hours of exposure to 70% ethanol is sufficient to kill established biofilm of gram-positive bacteria, gram-negative bacteria, and *Candida *spp. [95] and can successfully treat persistent bacteremia related to long-term intravascular devices [96]. Ethanol is effective in concentrations greater than 20%, and concentrations greater than 50% inhibit biofilm formation even if left in place for only 2 minutes [97]. No interactions with catheter structure have been reported with concentrations lower than 90%.

A first randomized, controlled trial of daily prophylactic lock solution instillation with a 2-hour dwell time compared 70% ethanol (34 patients) and heparinized saline (30 patients) in hematological patients with long-term catheters. Ethanol was associated with a decrease in CR-BSIs (9% [6/1,000 catheter-days] vs. 37% [31/1,000 catheter-days], *P *= 0.003) [98]. A recent randomized, controlled trial in 379 adult hematology patients failed to confirm these results (15 minutes daily of 70% ethanol lock solution, 0.7/1,000 catheter-days vs. control, 1.17/1,000 catheter-days; *P *= 0.22) [99]. Flushing the ethanol lock was associated with facial flushing (40%), altered taste (40%), and dizziness (50%) and should be strongly discouraged. A large French randomized, double-blind, controlled trial comparing a 2-min 60% ethanol lock to saline for hemodialysis catheters in ICU patients is ongoing and has already enrolled 1300 of the 1,560 planned patients (http://www.clinicaltrials.govNCT00875069).

## Conclusions

CR-BSI prevention relies chiefly on simple preventive measures and a continuous quality-improvement program. CR-BSI rates greater than 1 or 2 per 1,000 catheter-days are no longer acceptable. If the rate remains high under specific circumstances, or to lower the rate closer to zero, new devices or materials may help. Among these, only antiseptic-impregnated catheters and dressings have been proven effective to date. New materials or processes that prevent biofilm formation and bacterial growth are being tested. The efficacy and benefits of new preventive methods must be confirmed in ICUs where appropriate basal prevention is already optimal.

## Competing interests

JFT received consultancy fees from Carefusion and 3 M. JFT was a speaker at symposia organized by 3 M and Janssen-Cilag. JFT received research grants from Ethicon and 3 M. No other authors reported any potential conflicts of interest.

## Authors' contributions

CS made substantial contributions to the conception and design of the study, data acquisition, and data analysis and interpretation. JFT drafted the manuscript. All authors critically revised the manuscript for important intellectual content and approved the final version of the manuscript submitted for publication.
